# Supplementary material for: KGMP: Augmenting retrieval knowledge graph with multi-hop perceptron
Source: PLoS One. 2025 Oct 6;20(10):e0333037. doi: 10.1371/journal.pone.0333037 (PMC12500090; doi:10.1371/journal.pone.0333037)
Supplement: Attachment — Submitted filename: ReviewerComment.pdf [file pone.0333037.s001.pdf]

Abstract

1) KBQA faces a major challenge: queries often involve multi-hop questions, but existing KBQA methods lack sufficient higher-order subgraph information (please restructure this sentence, remove : in the sentence)
2) The experimental results show that by incorporating KGMP in ChatKBQA, the accuracy on WebQSP and CWQ datasets is increased by 6.2% and 5.3%, respectively → this quite confusing, study said it is working on Knowledge-Based Question Answering (KBQA), but then how KGMP is introduced in ChatKBQA.
3) Please highlight the study contribution in the abstract

Results and Discussion

Please add this section to discuss on research findings and contrbutions.
