# Supplementary material for: KGMP: Augmenting retrieval knowledge graph with multi-hop perceptron
Source: PLoS One. 2025 Oct 6;20(10):e0333037. doi: 10.1371/journal.pone.0333037 (PMC12500090; doi:10.1371/journal.pone.0333037)
Supplement: Attachment — Submitted filename: response letter(1).docx [file pone.0333037.s002.docx]

**Original Manuscript ID:** PONE-D-24-59861
**Original Article Title:** KGMP: Augmenting Retrieval Knowledge Graph with Multi-hop Perceptron

Dear Editor and Reviewers,

Thank you for the valuable comments to our manuscript entitled " KGMP: Augmenting Retrieval Knowledge Graph with Multi-hop Perceptron ". We have considered all the questions and suggestions from the reviewers and editor and revised the manuscript accordingly.

Enclosed please find a detailed response to the reviewers’ and editor’s comments. Comments (green), our response (black) and cited text from the revised manuscript (blue) are organized point by point. For the convenience of review, the modified contents in the revised manuscript have been highlighted.

Best regards,

Corresponding author: Duanbing Chen

# Response to Editor

## Comment 1**:**

*Please ensure that your manuscript meets PLOS ONE's style requirements, including those for file naming.*

Response:

We sincerely appreciate your valuable feedback and carefully revise the manuscript according to PLOS ONE’s Author Guidelines. Below we provide a point-by-point response to address the formatting requirements:

1. **Affiliation Formatting**
   We have restructured the author affiliations to comply with PLOS ONE’s specifications, removing paragraph formatting and ensuring proper vertical alignment. The zip codes have been systematically deleted from all institutional addresses as per the journal’s requirements.
2. **Figure Label Standardization**
   All figure references have been updated from “Figure” to the abbreviated form “Fig.”, aligning with PLOS ONE’s style manual.
3. **Author Contributions Statement**
   A new “Author Contributions” section has been added on page 18, utilizing the CRediT taxonomy to explicitly outline each author’s intellectual and technical contributions to the work.
4. **Funding Disclosure**
   The funding information has been integrated into the Acknowledgments section (page 18), including grant numbers and institutional support details, in compliance with PLOS ONE’s financial disclosure policy.
5. **Template Implementation**
   The manuscript has been fully reformatted using the official PLOS ONE LaTeX template (latest version, updated June 2022), ensuring complete adherence to the journal’s typographic standards and citation formatting requirements.

## Comment 2**:**

*we expect all author-generated code to be made available without restrictions upon publication of the work.*

Response:

Thank you for your valuable feedback regarding the code availability of the KGMP framework. However, we are sorry to say that we are unable to fully open-source the entire code. If readers are interested, they can contact the corresponding author or first author by email to obtain the core code for academic research. We have statemented this in the revised manuscript.

### Code availability

Source codes for the model described in this paper can be available upon request via email (zjyanguestc@163.com). The use of these codes is strictly limited to academic research purposes and requires proper authorization for any other applications.

## **Comment 3:**

*We note that the grant information you provided in the ‘Funding Information’ and ‘Financial Disclosure’ sections do not match.*

### **Response:**

We are so sorry for this mistake. We have implemented the following revisions in accordance with ICMJE recommendations and journal guidelines:

(a) Updated the **Acknowledgements** section (Page 16, lines 23-27) to explicitly state:
*“*This work was partially supported by the Key Research and Development Project of Sichuan under Grant No. 2024YFG0008 and the Major Program of National Natural Science Foundation of China under Grant No. T2293771.*”*

(b) Synchronously revised the **Financial Disclosure** statement in our submission system to include:
*“The authors have declared that no competing interests exist.”*

## **Comment 4:**

*Please amend your list of authors on the manuscript to ensure that each author is linked to an affiliation.*

### **Response:**

Thank you for highlighting the need for precise author affiliation mapping. We have revised the manuscript according to ICMJE authorship criteria and the journal’s affiliation policy:

**Affiliation Linkage Update**

In the manuscript’s title page (Page 1), we have:
• Added superscript numbering of affiliation after each author’s name established a clear correspondence between the author's name and the name of the affiliation. In the revised version, the affiliation of each author has been explicitly specified.

1) Big Data Research Center, University of Electronic Science and Technology of China, Chengdu, China
2) Chengdu Union Big Data Tech. Inc., Chengdu, China
3) Cuiying Middle School, Shantou, China
4) Suining Institute of Digital Economy, Suining, China

## **Comment 5:**

*Please upload a copy of Figure 5, to which you refer in your text on page 11.*

### **Response:**

We are so sorry for missing Figure 5 in the original submission. In the revised manuscript, this figure has been properly uploaded and integrated into the text. Specifically:

1. The content corresponding to the original Figure 5 appears as **Figure 6** in the revised manuscript (Page 14).
2. This numbering adjustment resulted from the insertion of a new figure (labeled Figure 5 in the revised manuscript) that better contextualizes the experimental setup.
3. All textual references to this figure have been systematically updated to reflect the new designation (Figure 6) throughout the manuscript.

**Fig 6. Comparison of average input Tokens length between ChatKBQA and ChatKBQA+KGMP.**

# Response to Reviewer #1

## **Comment 1:**

*While the proposed framework effectively addresses multi-hop queries, the mechanism for determining when additional subgraph information is needed could benefit from more detailed explanation.*

### **Response:**

We sincerely thank you for your insightful comments and constructive suggestions. In response to your concern about the mechanism of subgraph information introduction, we have systematically elaborated in the revised manuscript through the following two aspects:

1. Current KBQA methods perform well in handling single-hop queries, but have significant limitations when facing multi-hop reasoning scenarios. For example, ChatKBQA lacks of display modelling of high-order subgraphs, which leads to the problem of broken reasoning paths. This study innovatively introduces dynamic subgraph expansion to effectively enhance the capability of existing methods for multi-hop Q&A.
2. The decision mechanism for the depth and timing of subgraph expansion is dynamically decided by a fine-tuned LLM, whose decision-making capability is derived from pattern learning, and a large number of *<multi-hop problem, subgraph sequence>* training pairs constructed during the fine-tuning phase enables the model to establish a mapping relationship between the semantics of the problem and the subgraph order.

We list the revised contents as following for your convenient review.

### Determining Subgraph Information Sufficiency in LLMs

In the polling mechanism of KGMP, the core challenge lies in how LLMs dynamically determine subgraph expansion based on user query semantics and current subgraph topology. This section investigates the formation mechanism of LLMs’ decision logic, where the judgment capability primarily stems from two dimensions of knowledge internalization: historical pattern transfer learning and knowledge-driven state evaluation.

**Historical Pattern Transfer Learning:** Through fine-tuning with complete query path samples which consist of triplets representing a question, the corresponding subgraph state, and the expansion decision, LLMs acquire two critical decision patterns: (1) Initiate subgraph expansion when existing GQL query paths lack crucial semantic-associated edges; (2) Terminate expansion upon detecting minimal connected subgraphs sufficient for problem-solving.

**Knowledge-Driven State Evaluation:** LLMs’ capability to assess subgraph information adequacy derives from dual knowledge integration: (1) **Pretrained Knowledge Base:** Massive corpus pretraining establishes commonsense knowledge repositories, enabling deep semantic parsing of natural language instructions and providing cognitive frameworks for domain knowledge transfer; (2) **Domain-Specific Fine-Tuning:** Through structured GQL syntax samples and logical reasoning chain reinforcement, LLMs develop mapping capabilities between graph topology and problem semantics. For example, when processing the query “What electorate does Anna Bligh represent?”, the model executes the following reasoning chain: (a) The primary subgraph contains only the attributes of the *government.politician* entity and lacks associations with electoral districts; (b) Activates pattern matching mechanism to select *government_positions_held* relationship for expansion; (c) Detects *district_represented* attribute edge in second-order subgraph, confirming information completeness; (d) Terminates expansion and generates final answer.

This hierarchical knowledge architecture enables LLMs to dynamically evaluate subgraph states and autonomously optimize query paths, with decision-making essentially representing synergistic computation between pretrained knowledge generalization and domain-specific fine-tuning specialization.

## **Comment 2:**

*The paper could elaborate more on potential limitations of the KGMP module, such as the scalability of the subgraph retrieval process and its impact on performance with very large knowledge graphs. Additionally, what happens when there is no higher-order subgraph available? Does the model still return an answer, or does it fail gracefully?*

### **Response:**

Thank you very much for your constructive suggestions. We have analysed the limitations of the KGMP module in the revised manuscript. In fact, some key factors may affect the effectiveness and efficiency of the KGMP module. (1) Whether KGMP can obtain accurate subgraphs directly affects the accuracy of the final results. If KGMP obtains wrong subgraphs or there is no usable subgraph, the results tend to be incorrect. (2) The size of the knowledge graph will largely affect the performance of KGMP, because KGMP requests knowledge graphs multiple times in the retrieval process. The process time increases dramatically when KGMP retrieves on very large-scale knowledge graph. (3) The retrieval error rate of the LLM increases with the length of input sequences increasing since KGMP lead to a siginificant increase in input sequences. Besides, the increase of input sequences will also bring about great demand of computing power.

The revised contents are listed below for your convenient review.

### Subgraph mismatch

The subsequent error analysis demonstrated that 44% of errors are attributable to incorrect path selection. The path selection errors mainly come from following three factors: (1) improper selection of the direction of high-order subgraph expansion; (2) wrong relationship selection due to semantic bias in the graph query language generation process even though the subgraph is correct; and (3) premature termination of the traversal process when the expected subgraph size is not reached. It is worth noting that when a subgraph direction selection error occurs, the model terminates the traversal after a fixed number of steps even if the current subgraph does not contain the target answer. This property may be related to the inherent properties of the datasets - the fine-tuned data of KGMP comes from two datasets of WebQSP and CWQ, which have significant differences in the size of subgraphs involved in multi-hop queries, and the average number of traversals of KGMP is not the same on two datasets when reasoning, which is closely related to the distributional characteristics of query complexity between different datasets.

In order to further verify the model robustness, this study constructs a test set containing error subgraphs as well as subgraphs without high-order for validation experiments. The experimental results show that KGMP fails to derive the correct answers from the error subgraphs or subgraphs without high-order, which is directly related to the generation mechanism of its training data. Since the training samples of the model are all generated based on completely correct raw data, KGMP fails to acquire the inference pattern of the error subgraph or subgraph without high-order during the training process, resulting in its inference ability based on the correct knowledge subgraph only. This finding reveals the limitations of current knowledge graph inference models in terms of fault-tolerance mechanisms and provides an important direction for future study.

### KGMP performance on very large KG

In order to investigate the impact of knowledge graph size on KGMP performance, the database memory is constrained in order to simulate the situation of an extremely large knowledge graph being loaded. The query-answering time was split into two parts: (1) GQL execution time (largely affected by CPU traversal speed and the amount of available memory for indexing); and(2) LLM inference time (primarily running on GPU and not sensitive to CPU/memory constraints). As the size of the knowledge graph increased (i.e., the available memory decreased), a significant rise in GQL execution time was observed, leading to a substantial increase in overall query time. In contrast, LLM inference time remained almost constant throughout, primarily due to the fact that the reranker module prunes the candidate relations to a fixed number, thereby ensuring that the input to the LLM remains approximately the same size, regardless of the graph's overall scale. This phenomenon is further elucidated in Fig 7, which demonstrates that GQL execution time exerts a dominant influence on the overall performance cost in large graphs, while the LLM inference component remains relatively unimpacted.

**Fig 7. Comparison of LLM inference elapsed time and GQL execution spending time with different memory footprint reductions.**

### Retrieval performance degradation in long input sequences

In KBQA, multi-hop inference often requires traversing multiple subgraph levels, which leads to an exponential increase in the length of the input sequence. It is worth noting that Liu et al.[R1] empirically found that there is a significant "Lost in the middle" phenomenon in the knowledge retrieval process of large-scale language models, which is manifested in the following two aspects: (1) Document position sensitivity: When the document containing the correct answer is located at the beginning or end of the input sequence, the model retrieval accuracy can reach the peak. However, if it is in the middle of the sequence, the accuracy decreases by more than 20%, which shows obvious retrieval position bias. (2) Sequence length dependence: In the control variable experiments, when the input sequence lengths are 2k, 4k and 6k tokens, the model's retrieval accuracy for the first document shows a decreasing trend (77% → 75% → 72%), indicating that long sequence processing exacerbates the model performance decay. This finding has important implications for KGMP: the linear growth of input sequences and the nonlinear decay of model performance form a sharp contradiction when current architectures extend the depth of subgraph search. Therefore, how to construct an efficient sequence compression mechanism to reduce computational complexity while maintaining semantic integrity will become a key research direction to improve the effectiveness of multi-hop inference.

[R1] Liu, N., Lin, K., Hewitt, J., Paranjape, A., Bevilacqua, M., Petroni, F. & Liang, P. Lost in the middle: How language models use long contexts. Transactions of the Association for Computational Linguistics. 12 pp. 157-173 (2024)

## **Comment 3:**

*Missing references.*

### **Response:**

We would like to express our sincere apologies for the oversight in the citation. We have meticulously reviewed the manuscript and cited the relevant references, adding corresponding descriptions in the revised manuscript. For further details, we have added references regarding the analysis of knowledge graph-guided reasoning architectures[R2, R3, R4] and cited them in the section *KBQA methods* [Page 4]. [R2] is about *Think-on-graph 2.0* ( ref 34 in revised manuscript) and it is cited in Section KBQA methods (Page 4, line 13) to support the analysis of knowledge graph-guided reasoning architectures. [R3] is about *Retrieval-augmented generation* ( ref 35 in revised manuscript) and it is cited in Section KBQA methods (Page 4, line 15) to discuss QA system implementations. [R4] is about OneGen ( ref 36 in revised manuscript) and is cited in Section KBQA methods (Page 4, lines 16) to regard unified retrieval-generation frameworks. The corresponding descriptions are listed below for your convenience.

ToG-2[R2] aims to achieve a deep and comprehensive retrieval process by iteratively extracting information from both unstructured text and structured knowledge graphs. In contrast, Xu et al[R3] model the knowledge graph as a tree for question answering. Meanwhile, OneGen[R4] emphasizes the seamless integration of generation and retrieval.

[R2] Ma, S., Xu, C., Jiang, X., Li, M., Qu, H., & Guo, J. Think-on-graph 2.0: Deep and interpretable large language model reasoning with knowledge graph-guided retrieval. *ArXiv e-prints*, arXiv-2407.10805. (2024)

[R3] Xu, Z., Cruz, M. J., Guevara, M., Wang, T., Deshpande, M., Wang, X., & Li, Z. Retrieval-augmented generation with knowledge graphs for customer service question answering. In *Proceedings of the 47th International ACM SIGIR Conference on Research and Development in Information Retrieval*, pp. 2905-2909. (2024)

[R4] Zhang, J., Peng, C., Sun, M., Chen, X., Liang, L., Zhang, Z., Zhou, J. Chen, H. & Zhang, N. Onegen: Efficient one-pass unified generation and retrieval for llms. In *proceedings of the Association for Computational Linguistics: EMNLP 2024*, pp. 4088-4119. (2024)

# Response to Reviewer #2

## **Comment 1:**

*KBQA faces a major challenge: queries often involve multi-hop questions, but existing KBQA methods lack sufficient higher-order subgraph information (please restructure this sentence, remove : in the sentence)*

### **Response:**

We are so sorry for the confusion. We have rephrased this key statement as:

*“KBQA confronts significant challenges in addressing multi-hop questions, primarily due to the limited capability of existing methods to effectively leverage higher-order subgraph information.”*

## Comment 2:

*The experimental results show that by incorporating KGMP in ChatKBQA, the accuracy on webQSP and CWQ datasets is increased by 6.2% and 5.3%, respectively →this quite confusing, study said it is working on Knowledge-Based Question Answering (KBQA), but then how KGMP is introduced in ChatKBQA.*

### **Response:**

We are so sorry for confusing. In light of the limitations of ChatKBQA with regard to multi-hop questions, this study implements the subgraph traversal necessary for multi-hop reasoning by introducing KGMP as a subgraph retrieval module prior to ChatKBQA's execution of the S-expression generation process. Below, we clarify the integration mechanism of the KGMP module into the ChatKBQA:

1. **Role of KGMP in ChatKBQA**:
   KGMP serves as a **retrieval-augmented module** within ChatKBQA. Its primary function is to iteratively refine the retrieval of relevant subgraphs from the KG by modeling multi-hop reasoning paths. This addresses the inherent limitations of single-hop retrieval in complex KBQA scenarios.
2. **Integration Mechanism**:
   - During preprocessing, KGMP processes the input question and generates **optimized multi-hop subgraphs** by propagating entity-relation messages across the KG.
   - These subgraphs are then fed into ChatKBQA’s encoder-decoder architecture, enabling the model to focus on contextually relevant KG substructures while generating answers.

We have revised the abstract and added some notes to the text so as to clarify the role of KGMP in ChatKBQA. The revised version includes additional clarification to facilitate improved comprehension for readers [Page 5, Overview of KGMP subsection]. The corresponding revisions are listed below for your convenience.

### Abstract

The core challenge of Knowledge Base Question Answering (KBQA), as a bridge between natural language and structured knowledge, is to accurately map complex semantic queries into Graph Query Language (GQL). Compared with the traditional Text-to-SQL task, KBQA faces a dual challenge: the structural differences between GQL and SQL and the lack of higher-order subgraph information in multi-hop inference of knowledge graphs. While existing approaches such as ChatKBQA have made progress, the limitation of subgraph scalability severely constrains multi-hop query performance. To this end, this study proposes Knowledge Graph Multi-hop Perceptron (KGMP) - a retrieval-generation framework fine-tuned based on open-source large language models, whose innovativeness is reflected in three aspects: 1. **Dynamic Graph Traversal Mechanism:** Through an iterative subgraph expansion strategy, KGMP effectively achieves dynamic traversal of problem oriented graphs with progressive reasoning. 2. **Structured Interaction Protocol:** Based on SparQL syntax, KGMP designs a lightweight interaction instruction set to build an efficient communication interface between LLM and knowledge graph; 3. **Graph Structure Optimization Technique:** Develop subgraph reordering algorithms and pruning strategies based on the reranker model to ensure that the subgraphs input to the LLM are both compact and semantically complete. By integrating KGMP into the ChatKBQA framework and providing it with optimized multi-hop subgraph input, the experimental results show a performance improvement of 6.2% and 5.3% on WebQSP and CWQ datasets, respectively. This study provides a new technical paradigm for deep collaboration between LLM and knowledge graph.

### Overview of KGMP

KGMP is a specialized retrieval-generation KBQA framework tailored for KBQA, utilizing fine-tuning open-source LLMs. …

To perform the Q&A task, ChatKBQA first converts the input natural language query into a logical expression skeleton. It then fills this skeleton with specific content based on similarity metrics. However, when creating the logical skeleton, ChatKBQA only uses the query itself to estimate the hop count of relevant knowledge graph subgraphs, lacking detailed subgraph information. To overcome this limitation, KGMP provides the retrieved knowledge graph subgraphs and their retrieval processes to ChatKBQA as historical dialogue context. This enhances ChatKBQA’s understanding of subgraph structures, thereby improving the accuracy and completeness of answers.

## **Comment 3:**

*Please add “Results and Discussion” section to discuss on research findings and contrbutions.*

### **Response:**

Thank you for your valuable comment. We have carefully addressed your suggestion to add a dedicated “Results and Discussion” section. On the one hand, we have made some adjustments to the structure of the manuscript, and on the other hand, we have added some new elements to make the manuscript description clearer. For details, the key modifications are as follows:

1. **Structural Reorganization**
   - Relocated “Settings” from “Experiments results” section in the original manuscript to the “Method” section in the revised manuscript to enhance methodological completeness
   - Renamed the original “Experiments results” section to “Results and discussion” with expanded content
   - The “Results and discussion” section is now structured as follows: “**Main results**”, “**Validity analysis**”, “**Limitation analysis**” and “**Determining subgraph information sufficiency in LLMs**”.
2. **Additions to the Results and discussion section**

Introduced subsection “**Future directions**” discussing:

• Computational Resource Optimization and System Robustness Enhancement.

• Performance Limits in Ultra-Large-Scale Knowledge Graph Scenarios.

• Efficient Query Strategy Optimization.

The revised structure now better aligns with standard academic paper conventions while maintaining logical flow from methodology to evidence-based conclusions.

The revised contents are listed below for convienance review.

### Future Directions

In the subsequent research, the optimization of KGMP will focus on two core directions. Firstly, the dual goals of optimizing computational power consumption and improving robustness will be achieved by focusing on breaking through the key technology of effective error correction when the system makes mistakes during subgraph traversal under low computational power constraints. Secondly, the performance limits under the scenario of ultra-large knowledge graphs will be investigated, and the robustness improvement mechanism will be explored based on reinforcement learning. It is noteworthy that the optimization of query strategies to minimise interaction frequency with the database can be an effective means of enhancing the execution efficiency of KGMP in billion-scale ternary knowledge graphs.
